# Which health-related quality of life score? A comparison of alternative utility measures in patients with Type 2 diabetes in the ADVANCE trial

**DOI:** 10.1186/1477-7525-5-21

**Published:** 2007-04-27

**Authors:** Paul Glasziou, Jan Alexander, Elaine Beller, Philip Clarke, the ADVANCE Collaborative Group

**Affiliations:** 1 Centre for Evidence-Based Practice, Institute of Health Sciences, Oxford University, Oxford OX3 7LF, UK; 2 Queensland Clinical Trials Centre, University of Queensland, Australia; 3 School of Public Health, University of Sydney, Australia

## Abstract

**Background:**

Diabetes has a high burden of illness both in life years lost and in disability through related co-morbidities. Accurate assessment of the non-mortality burden requires appropriate health-related quality of life and summary utility measures of which there are several contenders. The study aimed to measure the impact of diabetes on various health-related quality of life domains, and compare several summary utility measures.

**Methods:**

In the ADVANCE (Action in Diabetes and Vascular Disease: Preterax and Diamicron MR Controlled Evaluation) study, 978 Australian patients with Type 2 diabetes completed two health-related quality of life questionnaires at baseline: the EQ-5D and the SF-36v2, from which nine summary utility measures were calculated, and compared. The algorithms were grouped into four classes: (i) based on the EQ-5D; (ii) using fewer items than those in the SF-12 (iii) using the items in the SF-12; and (iv) using all items of the SF-36.

**Results:**

Overall health-related quality of life of the subjects was good (mean utility ranged from 0.68 (±0.08) to 0.85(±0.14) over the nine utility measures) and comparable to patients without diabetes.  Summary indices were well correlated with each other (r = 0.76 to 0.99), and showed lower health-related quality of life in patients with major diabetes-related events such as stroke or myocardial infarction. Despite the smaller number of items used in the scoring of the EQ-5D, it generally performed at least as well as SF-36 based methods. However, all utility measures had some limitation such as limited range or ceiling effects.

**Conclusion:**

The summary utility measures showed good agreement, and showed good discrimination between major and minor health state changes. However, EQ-5D based measures performed as well and are generally simpler to use.

## Background

Many randomized control trials of therapies for Type 2 Diabetes now routinely include a generic health-related quality of life instrument administered to patients at baseline and during follow-up. The two most commonly used generic instruments are the EQ-5D [1] and the Short-Form 36 (SF-36) [2]. The EQ-5D has been used in the UKPDS [3], the Fenofibrate Intervention and Event Lowering in Diabetes (FIELD) study [4], and most recently the ADVANCE (Action in Diabetes and Vascular Disease: Preterax and Diamicron MR Controlled Evaluation) study [5]. The SF-36 has been used in the Lipids in Diabetes Study (LDS) [6], ADOPT [7] and on a sub-set of patients in both FIELD and ADVANCE.

While the generic instruments provide information that enables comparison of the health status of the study participants with other populations (e.g. comparisons of the SF-36 domain scores with norms for the general population), they are primarily used in the valuation of outcomes in economic evaluation. This typically involves use of predefined algorithms to convert item survey responses into a utility scale where 1.0 implies the patient is in perfect health and 0.0 is a health state equivalent to death. This is the most commonly used approach to estimate quality adjusted life years (QALYs) and provides a composite measure that aims to incorporate survival and health-related quality of life into a single index. QALYs are a particularly important outcome measure in the evaluation of therapies for a disease such as diabetes where complications, such as amputation, can have a considerable impact on overall health-related quality of life.

While many trials in diabetes now routinely include one or more generic health-related quality of life instrument there is no consensus as to either which instrument should be used, or which algorithm should be applied, to convert patient responses into utilities. In the case of the EQ-5D, traditionally the most commonly used algorithm was derived from a study involving individuals from the UK population [8]. However, recently a comparable study using a random sample of the United States population has becoming available [9], thereby providing researchers with an alternative set of utility for the same health states.

There are at least seven published algorithms [10,12-17] for converting either item responses, or summary scores, from the SF-36 into preference based utility measures. Most of these algorithms are based on subsets of questions from the SF-36. For example, the SF-6D uses only 11 items of the 36 items, and other algorithms, such as those developed by Lundberg and colleagues [10], are based on the shorter generic instrument, the SF-12 (which is based on 12 of the 36 questions of the SF-36). Clearly researchers who intend to measure the health related quality of life of people with diabetes face an array of choices regarding which generic instrument to use, and then which algorithm to employ.

The purpose of this study is to compare summary statistics of the estimated utility values produced by different algorithms for common complications of diabetes. In particular we are interested to see if there are systematic differences in both the absolute mean utility values and the deviations associated with each type of diabetes-related complication.

## Methods

### Study population

All patients included in this study were participating in the ADVANCE (Action in Diabetes and Vascular Disease: Preterax and Diamicron MR Controlled Evaluation) study. ADVANCE is a randomised 2 × 2 factorial trial in 11,140 normotensive patients with Type 2 diabetes comparing (i) intensive, gliclazide-based therapy or regular, guideline-based glucose control therapy in patients with Type 2 diabetes, and (ii) intensive blood pressure lowering based on a perindopril-indapamide combination or matching placebo [11]. The intensive gliclazide-based regimen aims to reduce haemoglobin A1C to 6.5% or lower (compared with haemoglobin A1C targets of 7–8% suggested by most regional guidelines) [5].

### Quality of life instruments

Two health-related quality of life questionnaires are being used in the ADVANCE trial: the EQ-5D (EuroQol 5-Dimensions) and the SF-36v2 (Short Form 36 version 2). The EQ-5D has five questions – on mobility, personal care, usual activities, pain/discomfort, and anxiety/depression – each encompassing the range of values 1 (= no problems), 2 (= some problems), 3 (= severe problems).

The SF-36v2 is a multipurpose short-form survey with 36 questions that measure eight health attributes using multi-item scales containing 2 to 10 items each. These attributes are: (1) physical functioning, (2) role limitations due to physical health problems, (3) bodily pain, (4) general health, (5) vitality (energy/fatigue), (6) social functioning, (7) role limitations due to emotional problems, and (8) mental health (psychological distress and psychological well-being). The SF-12 and SF-6D are health-related quality of life instruments that use a subset of these items.

The EQ-5D was administered to all patients in the ADVANCE trial, from the 20 participating countries. The SF-36v2 questionnaire was administered to the subgroup of Australian patients only. Both questionnaires were administered at baseline and 2 years after randomization to the study. As the analysis involves a comparison of the EQ-5D with the SF-36 we have restricted the analysis to the Australian patients.

### Utility measures

We undertook a literature search to identify studies that reported algorithms to convert generic health-related quality of life instruments into utility values which could potentially be used in valuing outcomes in economic evaluations. We identified seven studies [10,12-17] that used items from the SF-36, and two [8,9] using the EQ-5D, that were published prior to December 2005. The main features of these algorithms (which are ranked by the number of items they require) are summarized in Table 1. The algorithms can be grouped into four classes: (i) those based on the EQ-5D [8,9]; (ii) those algorithms using fewer items than the SF-12 (i.e. SF-6D) [12,13] (iii) those that use the SF-12 [10,14-16]; (iv) those that use all items of the SF-36 [17]. All studies except Lawrence [14] and Franks [15,16] involve utilities derived from studies involving primary direct assessment of utilities e.g., using standard gamble or time trade-off methods.

**Table 1:** Algorithms to convert item responses from generic health-related quality of life instruments into utility measures

**Study (denoted by primary author and instrument)**	**Dolan EQ-5D**	**Shaw EQ-5D**	**Brazier SF-6D (SF-12)**	**Brazier SF-6D (SF-36)**	**Lundberg SF-12**	**Lawrence SF-12 (MEPS)**	**Franks SF-12 (MEPS)**	**Franks SF-12**	**Fryback SF-36**
**Type of Algorithm**	EQ-5D based algorithm		SF-6D based algorithm		SF-12 based algorithm				SF-36 based algorithm
**QoL Questionnaire**	EQ-5D	EQ-5D	SF-12	SF-36	SF-12	SF-12	SF-12	SF-12	SF-36
**Number items used**	5	5	7	11 (of 36)	12 (of 36)	12	12	12	36
**Methods for valuing utility**	Time Trade Off	Time Trade Off	Standard Gamble	Standard Gamble	Time Trade Off	Mapping to the UK based EQ-5D tariffs	Mapping to the UK based EQ-5D tariffs	Mapping to the UK based EQ-5D tariffs	Quality well being index
**Reported range in the ADVANCE trial patients**	-0.18 to 1	0.20 to 1	0.41 to 1	0.35 to 1	0.47 to 0.98	0.20 to 1.01	0.046 to 0.975	-0.07 to 0.94	0.509 to 0.836
**Health states sampled**	243	243	249	249	NA	NA	NA	NA	NA
**Population**	General population	General population	General population	General population	General population	General population	General population	Low income, minority population	General population
**Country where developed**	UK	USA	UK	UK	Sweden	USA	USA	USA	USA
**Description of process**	3,667 individuals rating 12 health states from the possible 243 states.	4,048 individuals rating 13 from the possible 243 states.	611 individuals rating 6 health states from 6-dimensional health state classification (3518 observations)	611 individuals rating 6 health states from 6-dimensional health state classification (3518 observations)	Postal questionnaire 5,400 individuals using SF12 plus TTO question. Regression used to derive utilities.	14,580 respondents who completed the Medical Expenditure Panel survey 2000.	15,000 respondents who completed the Medical Expenditure Panel survey 2000.	240 respondents attending a community health centre in New York	1,356 respondents who participated in the Beaver Dam Health study

### Statistical analysis

We report summary statistics for patients with a history of any of seven diabetes-related complications: stroke or transient ischemic attack (TIA), peripheral revascularization and/or amputation, hospital admission for unstable angina, myocardial infarction, coronary artery bypass graft, and currently treated for hypertension. This history was reported on the baseline form. The mean (sd) utility for those with the complication, without the complication, and without any of the complications, was calculated for all nine algorithms. Confidence intervals were calculated for differences in the mean level of utility of those experiencing each type of complication with those not experiencing that complication in the Australian ADVANCE population, and displayed graphically. To compare how the mean difference in utility varied between algorithms we ranked the severity (using mean deficit in utility values) of the seven complications of diabetes for each utility measure, then used Spearman's rank correlation coefficient to assess the degree of consistency in ranking. We also graphed the average difference in mean utility for these complications by the four classes of algorithm.

To estimate the within-person variability, we used the measures in the subgroup of patients who had had no major adverse event between the baseline and two-year qol measurements. This was used to define a signal-to-noise ratio as the difference in mean utility between those who had a major event and those who did not, divided by the standard deviation of the utility measure in those who did not have a major event. Furthermore, linear regression models were used to assess the statistical significance of the presence of a major event on change in 2-year utility score, adjusted for baseline score.

## Results

### Subjects

Characteristics of the ADVANCE study population have been published [11], and included a total of 978 Australian patients randomized. This Australian cohort was 71% male, had a mean age of 67 (range = 55–86) years at registration, and a baseline mean hemoglobin A1c (HbA1c) of 7.2 (sd = 1.2). The average duration of diabetes was 7.2 years (sd = 6.1 years). The two health-related quality of life measurement instruments, the SF-36v2 and the EQ-5D, were administered at baseline to all 978 patients, with 975 responses (99.6%) to the EQ-5D, and 978 responses (100%) to the SF-36v2.

### Utility scores

Table 1 includes information on the range of utility values in our study population. The Weighted Health Index (WHI) utility measure (calculated from the EQ-5D) was -0.18 to 1.0 when calculated using the Dolan algorithm, and 0.20 to 1 for the Shaw algorithm. The range of the Brazier index, based on the SF-6D, was 0.35 to 1.0, and the Lundberg 0.43 to 0.98.

**Figure 1:**
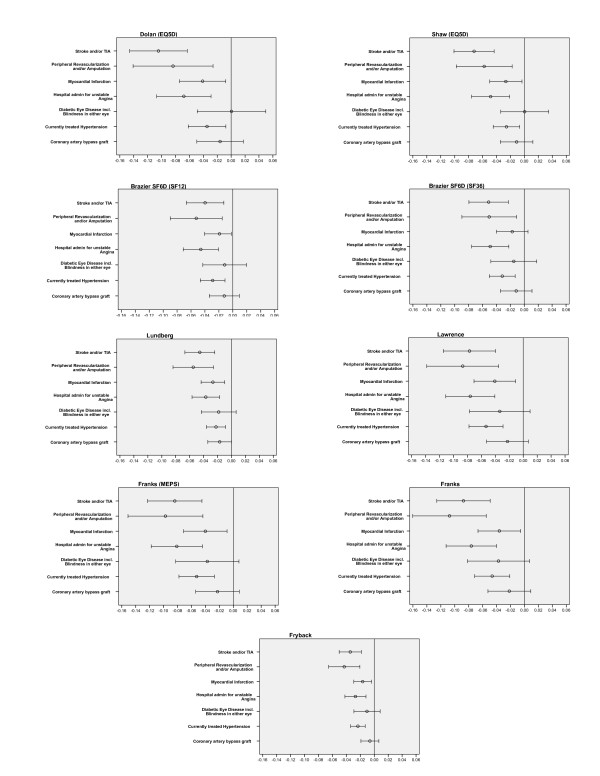
Mean deficit (and 95% CIs) in utility value at study baseline for patients with selected medical condition.

Table 2 shows the mean (sd) utility scores for each of the utility measures broken down by presence or absence of the seven individual diabetes-related complications at baseline. It also includes these same measurements for the subset of patients (21.5%) who had no history of any of these complications at baseline. There was moderate variation in the average utility values. For example, for the 10.5% of patients who had previously experienced a stroke or TIA, the mean utility ranged from approximately 0.650 (for Fryback and Franks SF-12-MEPS algorithms), up to 0.783 for the Shaw (EQ-5D) algorithm. When examining the difference in utility values between those who had a history of the nominated medical conditions, and those who didn't, the instruments based on either the EQ-5D (Dolan and Shaw), or based on instruments that mapped utility values from the EQ-5D (i.e. Franks MEPS, Lawrence MEPS, Franks SF-12) appear to have greater differences than the algorithms designed specifically for the SF-36 (i.e. Lundberg, SF-6D and Fryback). For example, the difference between the utility value of a past stroke/TIA, for those instruments based on EQ-5D or mapped values from the EQ-5D, ranged between a decrement of 0.104 (Dolan) to 0.073 (Shaw), while the decrement for the others ranged from 0.051 for Brazier (SF-36) to 0.034 for Fryback. Diagramatically, this information is presented in Figure 1, where, for each algorithm, the mean differences (and 95% CI) for all of the complications are shown as deficits in utility value, with zero indicating no difference.

**Table 2:** Mean (sd) utility measures by presence of diabetes-related complications at baseline

	**% of study population**	** Utilities **								
		**Dolan (EQ-5D)**	**Shaw (EQ-5D)**	**Brazier SF-6D (SF12)**	**Brazier SF-6D (SF36)**	**Lundberg (SF-12)**	**Lawrence (SF12-MEPS)**	**Franks (SF12-MEPS)**	**Franks (SF12)**	**Fryback (SF36v1)**
**All patients**	100% (n = 975)	0.801 (0.206)	0.848 (0.144)	0.780 (0.131)	.746 (0.138)	0.801 (0.103)	0.728 (0.182)	.725 (0.189)	0.744 (0.186)	0.678 (0.079)
**MEDICAL CONDITION**										
Stroke and/or TIA	Y 10.5%	.708 (0.259)	.783 (0.174)	.744 (0.141)	.700 (0.142)	.762 (0.112)	.659 (0.192)	.650 (0.207)	.666 (0.211)	.648 (0.082)
	N	.812 (0.196)	.856 (0.138)	.784 (0.129)	.751 (0.136)	.804 (0.102)	.736 (0.179)	.734 (0.185)	.754 (0.181)	.682 (0.078)
Peripheral Revascularization and/or Amputation	Y 5.4%	.722 (0.236)	.793 (0.159)	.730 (0.149)	.698 (0.146)	.751 (0.116)	.646 (0.207)	.633 (0.232)	.643 (0.250)	.638 (0.078)
	N	.805 (0.204)	.851 (0.143)	.782 (0.129)	.749 (0.137)	.803 (0.102)	.733 (0.179)	.730 (0.185)	.750 (0.180)	.681 (0.079)
Hospital admin for unstable Angina	Y 12.6%	.741 (0.242)	.805 (0.165)	.739 (0.148)	.703 (0.146)	.768 (0.116)	.661 (0.198)	.654 (0.214)	.678 (0.222)	.655 (0.083)
	N	.809 (0.199)	.854 (0.140)	.785 (0.127)	.752 (0.137)	.804 (0.101)	.737 (0.177)	.735 (0.184)	.754 (0.179)	.682 (0.078)
Myocardial Infarction	Y 18.8%	.767 (0.223)	.826 (0.152)	.764 (0.136)	.732 (0.138)	.778 (0.105)	.695 (0.182)	.692 (0.195)	.715 (0.200)	.665 (0.078)
	N	.808 (0.202)	.853 (0.142)	.783 (0.130)	.749 (0.138)	.805 (0.103)	.736 (0.181)	.732 (0.188)	.751 (0.182)	.682 (0.079)
Coronary artery bypass graft	Y 18.6%	.788 (0.211)	.839 (0.146)	.769 (0.136)	.736 (0.140)	.788 (0.107)	.710 (0.185)	.706 (0.198)	.727 (0.205)	.673 (0.082)
	N	.804 (0.205)	.850 (0.144)	.782 (0.130)	.748 (0.137)	.803 (0.102)	.732 (0.181)	.729 (0.187)	.748 (0.182)	.680 (0.079)
Currently treated Hypertension	Y 64.7%	.789 (0.209)	.839 (0.145)	.769 (0.132)	.735 (0.137)	.794 (0.102)	.710 (0.181)	.707 (0.190)	.728 (0.189)	.670 (0.078)
	N	.823 (0.199)	.865 (0.141)	.798 (0.127)	.767 (0.138)	.814 (0.103)	.763 (0.178)	.759 (0.184)	.775 (0.177)	.694 (0.080)
Diabetic Eye Disease incl. Blindness in either eye	Y 7.6%	.801 (0.177)	.848 (0.126)	.769 (0.131)	.732 (0.142)	.779 (0.105)	.697 (0.190)	.690 (0.202)	.710 (0.199)	.669 (0.073)
	N	.801 (0.208)	.848 (0.145)	.780 (0.131)	.747 (0.138)	.801 (0.103)	.731 (0.181)	.728 (0.188)	.747 (0.185)	.679 (0.080)

**Patients without above conditions at baseline**	21.5%	.843 (0.186)	.877 (0.135)	.802 (0.125)	.771 (0.137)	.824 (0.098)	.778 (0.174)	.774 (0.177)	.788 (0.165)	.701 (0.079)

**Duration of Diabetes**	>= 5 yrs 47.3%	.808 (0.208)	.853 (0.146)	.782 (0.133)	.750 (0.141)	.796 (0.105)	.726 (0.185)	.722 (0.193)	.740 (0.191)	.678 (0.082)
	6+ yrs 52.7%	.793 (0.205)	.843 (0.142)	.777 (0.129)	.743 (0.135)	.802 (0.102)	.730 (0.179)	.727 (0.186)	.748 (0.182)	.679 (0.077)

**Figure 2:**
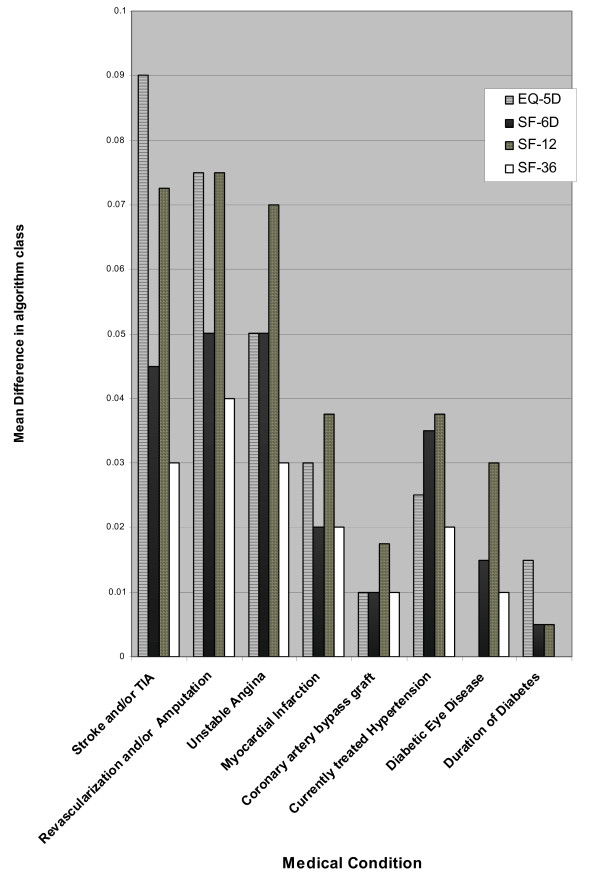
Difference in mean utility for selected complications (grouped by 4 classes of algorithms).

Table 3 shows the results of the rank correlations of the utility differences derived from values reported in Table 2. Overall there is a good consistency in the ranking for the seven complications: the Spearman's rank correlation coefficient ranges from 0.991 (between Shaw and Dolan) to 0.757 (between Dolan and Franks). The utility differences were then allocated to the four algorithm classes and averaged for each class. Figure 2 graphically represents the variability amongst the four algorithm classes.

**Table 3:** Correlations between utility measures on ranking of severity of seven complications of diabetes

**UTILITY MEASURES**	**Dolan**	**Shaw**	**Brazier SF6D (SF12)**	**Brazier SF6D (SF36)**	**Lundberg**	**Lawrence**	**Franks MEPS**	**Franks**	**Fryback**
**Dolan**	1.000								
**Shaw**	0.991*	1.000							
**BrazierSF6D (SF12)**	0.837	0.862	1.000						
**BrazierSF6D (SF36)**	0.842	0.878	0.943	1.000					
**Lundberg**	0.852	0.860	0.868	0.932	1.000				
**Lawrence**	0.917	0.925	0.953	0.971	0.932	1.000			
**Franks_MEPS**	0.818	0.853	0.944	0.972	0.962	0.943	1.000		
**Franks**	0.757	0.791	0.835	0.935	0.972	0.878	0.963	1.000	
**Fryback**	0.918	0.926	0.963	0.923	0.933	0.962	0.953	0.871	1.000

* Spearman's rank correlation coefficient

The two health-related quality of life instruments (SF-36 and EQ-5D) were administered to all patients again at approximately two year after baseline. During this time, details of any serious adverse events (SAEs) experienced by the patients were also collected and then used in conjunction with the 2-year qol information to examine changes in health status since baseline. In the ADVANCE study, serious adverse events are defined as events that: (1) result in death, (2) are life threatening in the opinion of the responsible investigator,(3) require inpatient hospitalisation or prolongation of existing hospitalization, (4) result in persistent or significant disability or incapacity, (5) result in congenital anomaly or birth defect (unlikely since participants must be aged 55 years or over at entry to study), and (6) are important medical events in the opinion of the responsible investigator. The presence or absence of an SAE was then used in conjunction with the difference in utility measure between baseline and two-year values to calculate regression coefficients and signal/noise ratios (Table 4). Most algorithms showed similar signal-to-noise ratios, indicating that they have similar sensitivity to clinically relevant changes in health status.

**Table 4:** Changes in utility measures at 2 yrs by presence or absence of Serious Adverse Event episodes

Serious Adverse Event episodes (in 2-year period)		Dolan	Shaw	Brazier SF6D (SF12)	Brazier SF6D (SF36)	Lundberg	Lawrence	Franks MEPS	Franks	Fryback
**NO**	Mean difference*	-.024	-.017	-.019	-.019	-.021	-.023	-.022	-.018	-.013
	Std. Deviation	.196	.139	.115	.121	.078	.142	.146	.146	.068
**YES**	Mean difference*	-.042	-.028	-.035	-.031	-.037	-.046	-.052	-.058	-.015
	Std. Deviation	.230	.157	.127	.125	.087	.156	.173	.190	.068
Regression coeff** (P value)		-.040 (.003)	-.028 (.003)	-.033 (<.001)	-.032 (<.001)	-.024 (<.001)	-.046 (<.001)	-.053 (<.001)	-.062 (<.001)	-.017 (<.001)
Signal/Noise ratio		.090	.082	.136	.104	.202	.161	.205	.277	.037

Note1: * Mean difference is the mean of the difference between the two-year and baseline values for each utility measure.

Note2: ** Regression model assessed significance of Serious Adverse Event episodes on difference in 2 yr score, adjusted for baseline score.

## Discussion

Health technology assessments increasingly rely on generic health-related quality of life instruments, and published algorithms, for deriving utilities in order to quantify outcome of interventions to prevent or treat diabetes and its complications. This study provides evidence on the degree of variation in utility scores for common diabetes-related complications when they are derived using different algorithms for patients enrolled in the ADVANCE trial.

Our results demonstrate that there is a moderate variation among algorithms in the average utility score for many complications, and in the degree to which those with complications differ from the rest of the ADVANCE patients. While there is a high level of agreement among the instruments in their ranking of the severity of complications, it would appear that those algorithms based on the EQ-5D produce greater mean differences for macro vascular complications such as stroke, as might be clinically expected. For example, mean difference in utility for patients with a stroke or TIA compared with the rest of the ADVANCE patients is around 0.1 for the Dolan (EQ-5D) algorithm, but only 0.05 for the Brazier SF-6D (SF-36) algorithm (see Figure 1), but both algorithms rank it as having the greatest impact on the mean difference in utility.

The use of alternative utilities for the same health states when evaluating interventions for diabetes has important implications for cost-effectiveness analysis, as the degree to which a therapy may be cost-effective may depend on which method is used to derive utilities [18]. In these circumstances, what criteria could be used to choose an appropriate method for assessing health-related quality of life of patients with diabetes? One obvious approach would be to compare the utilities derived from these generic instruments with those elicited directly from patients, or the community, using such methods as time-trade-off or standard gamble techniques. Unfortunately, while directly elicited utility values have been reported for a limited range of complications (e.g. amputation [18]), currently there is insufficient information to undertake a systematic comparison.

Secondly, the utility algorithm should provide sufficient sensitivity. This includes both being responsive to major changes in health state (see criterion 1 above), and also demonstrating minimal day-to-day fluctuation when no major changes have occurred. That is, a good "signal-to-noise" ratio. Those instruments based on direct valuation of health states (i.e. EQ-5D and SF-36) tend to have lower signal to noise ratios (indicating greater variation in the utility measures) than those using some form of mapping, however this is due to differences in methods of derivation. In particular, the latter is generally based on predictions of mean utilities derived from regression equations that tend to reduce the degree of variation across health states. In regard to the instruments based on direct valuation, the EQ-5D generally seemed to perform as well as the SF-36 based methods. This is somewhat surprising given the smaller number of items assessed and used in the scoring of the EQ-5D.

A third criterion for choosing a utility instrument is the generation of an appropriate range for the utility values. In this regard, the Brazier algorithm, which is now the standard summary score for the SF-36, has a much narrower range as it cannot achieve scores lower than 0.296. This limits the degree to which it can characterize the utility associated with extremely poor health states, such as disabling stroke. The Lundberg score was highly correlated with the Brazier score and showed similar problems of range restriction. This may explain why these algorithms generally produced smaller differences for those with and without particular complications, than algorithms based on EQ-5D utility values. This has also been observed in other disease areas such as patients with liver transplantation [19] in which patients 12 months after transplant had a significant improvement in utilities derived from the EQ-5D, but not from the SF-6D. A similar pattern has been found for patients visiting a rheumatology clinic [20].

A final criterion is the simplicity or ease of administration of the instrument. The collection of health-related quality of life information is often subject to cost or time constraints, as the contact time with a patient in trials is limited. Hence the administration of the health-related quality of life instrument has an opportunity cost in terms of reducing the time available to collect other information. The SF-36 is usually administered as a self-completed questionnaire which contains 36 items covering different aspects of health-related quality of life, and administered when patients attend clinics. While the developers of the SF-36 state that it can usually be completed in 5 to 10 minutes[21], at least one study has indicated that it may take, on average, 15 minutes to administer in some elderly populations [22]. The EQ-5D is a shorter five item questionnaire which has three response levels to each item and hence its administration time is likely to be well below five minutes. Besides the cost of administration, the use of the EQ-5D may have other advantages as it has been shown to have a higher response rate than those based on the SF-36 and, given the small number of items, there is a greater chance of full-completion which minimizes the problem of missing data [23]. Unless the researcher is interested in deriving domain scores for the SF-36, another option would be to ask a sub-set of questions that match the existing instruments. In this regard, the SF-12 would appear to be a good choice, as five of the seven algorithms were based on SF-12.

Since researchers must choose a utility algorithm in addition to choosing a quality of life instrument, the differences in the utilities for the EQ-5D derived using the algorithms developed by Shaw for a US population and original tariffs that were derived in the United Kingdom are of note. For example, the Dolan algorithm indicated patients with no complications had a utility around 0.8 (similar to previous studies that used this algorithm [3,24]), while the Shaw algorithm utility gave values 0.05 higher for the same group of patients. While this may reflect differences in health related quality of life between these populations, it is not obvious which should be used, especially in countries where valuation exercises to derive tariff values for EQ-5D states have not been undertaken. Given the increasing internationalization of clinical trials it is important to address this issue in future work.

Finally it is important to consider the limitations of the study. First, patients were restricted to Australian patients with diabetes who were eligible and willing to participate in a clinical trial, and hence it may not be a representative sample. However, we were not trying to estimate population values but rather to analyze the relationship between health states and utility, and there appeared to be sufficient diversity of the sample for this. Second, and more crucial, was the limited data on the nature and timing of the prior and intercurrent events. For example, though we have treated stroke as a single state, its effects are diverse ranging from full recovery to severe disability. Third, we ignore multiple morbidities within individual patients, but as these were relatively rare it is unlikely to make much difference to the comparisons. Fourth, while we had patients complete the full SF-36, some of the utility scorings only used a subset of questions. Finally, our estimate of test-retest stability is based on patients with no event re-measured after 2 years, and it is likely that the variability represents measurement error plus some real, though minor, changes. However, this is common across all the utility measures and hence should not greatly influence the relative signal-to-noise ratio.

## Conclusion

In conclusion, there was considerable variation across the different algorithms for translating responses to the EQ-5D and SF-36 into utilities. Algorithms based on survey instruments covering more comprehensive aspects of health-related quality of life did not appear to measure greater variations in utility than those based on simpler instruments such as EQ-5D. Taking this into account, the lower administration time of the EQ-5D suggests that it has real advantages over the SF-36 when collecting health-related quality of life information as part of a clinical trial.

## Abbreviations

ADVANCE Action in Diabetes and Vascular Disease: Preterax and Diamicron MR Controlled Evaluation

UKPDS United Kingdom Prospective Diabetes Study

## Competing interests

The author(s) declare that they have no competing interests.

## Authors' contributions

PG conceived of the study, and drafted the manuscript. PC contributed to both the design of the study and the final manuscript. EB assisted in the statistical analyses and contributed to the final manuscript. JA carried out the data analyses and contributed to the draft of the manuscript and the final manuscript. All authors read and approved the final manuscript.
